# Relationship between knee and ankle degeneration in a population of organ donors

**DOI:** 10.1186/1741-7015-8-48

**Published:** 2010-07-28

**Authors:** Carol Muehleman, Arkady Margulis, Won C Bae, Koichi Masuda

**Affiliations:** 1 Department of Biochemistry, Rush University Medical Center, Cohn Research Building, 1735 W. Harrison, Chicago, IL 60612, USA; 2 Department of Radiology University of California, San Diego, 11379 Cadence Grove Way, San Diego, CA 92130, USA; 3 Department of Orthopaedic Surgery, University of California, San Diego, 11379 Cadence Grove Way, San Diego, CA 92130, USA

## Abstract

**Background:**

Osteoarthritis (OA) is a progressive degenerative condition of synovial joints in response to both internal and external factors. The relationship of OA in one joint of an extremity to another joint within the same extremity, or between extremities, has been a topic of interest in reference to the etiology and/or progression of the disease.

**Methods:**

The prevalence of articular cartilage lesions and osteophytes, characteristic of OA, was evaluated through visual inspection and grading in 1060 adult knee/tali pairs from 545 cadaveric joint donors.

**Results:**

Joint degeneration increased more rapidly with age for the knee joint, and significantly more knee joints displayed more severe degeneration than ankle joints from as early as the third decade. Women displayed more severe knee degeneration than did men. Severe ankle degeneration did not exist in the absence of severe knee degeneration. The effect of weight on joint degeneration was joint-specific whereby weight had a significantly greater effect on the knee. Ankle grades increasingly did not match within a donor as the grade of degeneration in either the left or the right knee increased.

**Conclusions:**

Gender and body type have a greater effect on knee joint integrity as compared to the ankle, suggesting that knees are more prone to internal causative effects of degeneration. We hypothesize that the greater variability in joint health between joints within an individual as disease progresses from normal to early signs of degeneration may be a result of mismatched limb kinetics, which in turn might lead to joint disease progression.

## Background

Osteoarthritis (OA) is a generally progressive condition that involves both anabolic and catabolic mechanisms within the articular cartilage and bone of synovial joints in response to both internal and external factors. Among these factors are age [1,2] genetics [3], joint/limb alignment [4-8], joint injury [9], female gender [10] and obesity [11,12]. On the other hand, exercise and local muscle strengthening can inhibit its progression [13,14] by strengthening the local environment and thus reducing instability at the joint. But because there is no cure for OA and because its etiology is not fully understood, investigation continues to further elucidate the mechanisms which may contribute to its initiation and progression.

Limb alignment and the relationship between the biomechanics as well as the incidence/prevalence of OA in one joint of an extremity in relationship to another joint within the same extremity has been a topic of reemerging interest. Recently, a cross-sectional study [15] found multiple kinematic and kinetic differences at the hip, knee and ankle joints in those individuals with severe knee OA. Furthermore, using mechanical axis radiographs, Tallroth et al. [16] found that the greater the tilt (relative angle of the talus to the distal tibia and distal fibula) in the ankle, the more degenerative were the changes.

Previously, in a sample of 50 knee and ankle donors, it was shown that donors with degeneration in the ankle also showed degenerative changes within the knee at an equal or higher level [17]. The data suggest that factors such as altered mechanics as a result of limb alignment might contribute to degeneration within an entire limb. Furthermore, although genetic factors might be involved in global joint degeneration within an individual, mechanical factors within one limb likely influence the joint health of the contralateral limb.

The goal of the present study was to expand our previous database [17] and correlate knee and ankle cartilage OA scores in an effort to further elucidate the relationship between degenerative joint disease within a limb and between limbs of an individual. We hypothesize that OA in one joint is associated with increased prevalence of OA in another kinematically related joint, with this relationship increasing with the severity of the OA.

## Methods

One thousand sixty adult knee/tali pairs were obtained from 545 joint donors through collaboration with the Gift of Hope Organ and Tissue Donor Network. This included two knees and two ankles from each cadaveric donor with the exception of 30 donors for whom only one lower extremity was available because the other was present but not available to our laboratory. The joints were collected between June 1995 and April 2009 according to the policies of the Gift of Hope and with Rush University Institutional Review Board approval. Exclusion criteria included previous amputation of an extremity, joint replacement in either lower extremity, a history of hepatitis or a postmortem positive blood test for hepatitis, HIV, or any other communicable disease. The distal portion of the tibia (proximal component of the ankle) was not available; therefore, the talus represents the ankle joint in this study. Although donor medical histories were not available, age, gender and cause of death were provided. The donors were categorized as light, normal or obese on the basis of the subjective visual assessment of the joint harvester.

**Figure 1:**
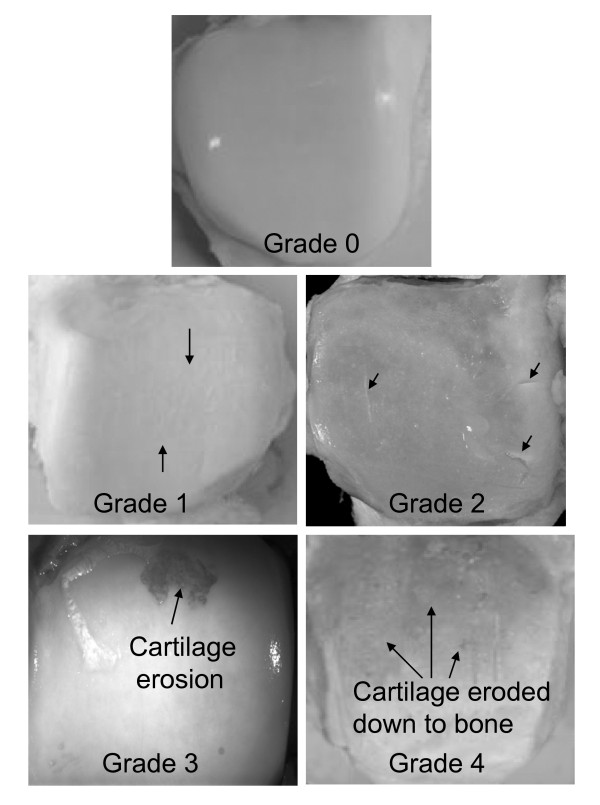
**Representative tali**. Examples of joint degeneration grades: **(a) **grade 0, **(b) **grade 1, **(c) **grade 2, **(d) **grade 3, **(e) **grade 4.

The joints were opened within 24 hours of death of the donor and examined for disruption of the articular cartilage on a modified Collins scale [18] where grade 0 is normal smooth cartilage, grade 1 is superficial fibrillation, grade 2 is fissuring or superficial ulceration with possible osteophytes, grade 3 is 30% or less of the cartilage surface eroded down to subchondral bone and accompanying osteophytes, and grade 4 is more than 30% of cartilage eroded down to subchondral bone with gross geometric changes including osteophytes (Figure 1). For knee cartilage scores, the highest (i.e., worst) score observed on the femur, patella, or tibia was taken as the score for the joint.

The following statistical analyses were performed for nonparametric data. Spearman's rank correlation was carried out to determine the correlation between age and joint degeneration for each joint individually. The Wilcoxon signed rank test was used to determine the relationship between the grade of left and right sides for ankle and knee joints separately. To determine the effects of tissue type (ankle vs. knee) and sex on age-dependent degeneration, survivor analysis was performed. The analysis determined the incidence (as survival rate) of the selected grades (set at 1 through 4) with increasing age (i.e., "survival curve"), stratified by tissue type. The mean survival age, the age at which half of the samples became degenerate (i.e., became the selected grade), was also determined. The survival curves were compared using Kaplan Meier analysis and Mantel statistic [19]. Additionally, the effect of sex on survival curves was determined separately for each tissue type. Statistical significance was taken at *P *< 0.05.

## Results

**Figure 2:**
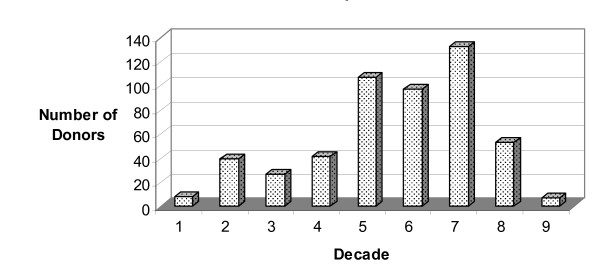
**Distribution of knee/tali donors per decade of life**. Mean age = 60 years.

Donors ranged in age from 19 to 98 with a mean age of 60 years. The age distribution per decade is shown in Figure 2. There were 287 (53%) men and 258 (47%) women. This profile reflects the donor population of the Gift of Hope Organ and Tissue Donor Network.

Within the current study population, joint degeneration was first observed during the third decade of life, starting with a 26-year-old male with both knees displaying fibrillated cartilage (grade 1). The earliest signs of degeneration in the ankles were in a 28-year-old male whose knees displayed grade 2 and ankles displayed grade 1 degeneration. The oldest donor, a 96-year-old female had knees with grade 4 degeneration and ankles with grade 2 degeneration.

**Figure 3:**
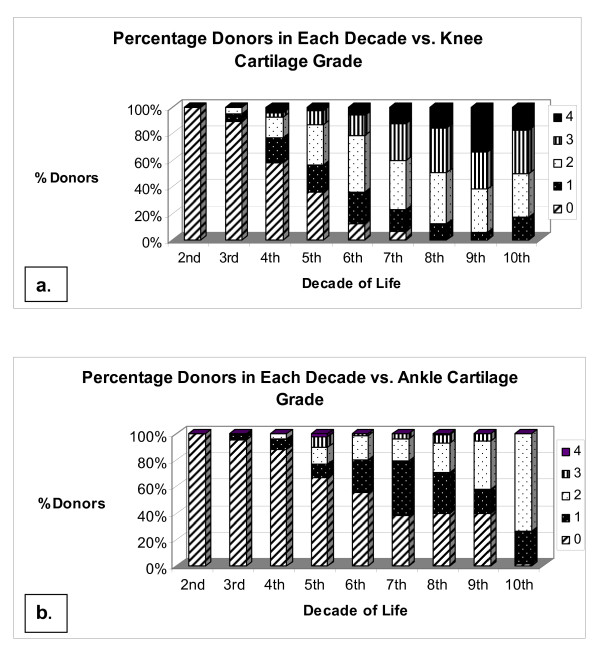
**(a) and (b) Distribution of grades per decade for **(a) **knee and **(b) **ankle joints**. By the third decade, the severity of knee grades increased in comparison to the ankle. By the eighth decade, no knee was normal in appearance, whereas some normal tali were observed into the tenth decade.

The percentage of donors in each decade displaying each grade of degeneration for knees and ankles is shown in Figures 3a and 3b, respectively. We found that 17% of individuals had both normal (grade 0) knee and ankle cartilage. Degeneration increased for ankles through the tenth decade. Spearman's rank correlation for the association between age and the level of degeneration in both left and right ankle and knee joints was statistically significant (*P *= 0.0001 for both joints). Degeneration increased for knees through the ninth decade, with a slight decrease in the tenth decade, but it should be noted that there were only six donors in the tenth decade. Degeneration increased more rapidly with age for the knee joint, and significantly more knee joints displayed more severe degeneration than ankle joints from as early as the third decade. A minority (4%) of knee joints displayed completely normal cartilage from the sixth decade upward, and no single knee joint displayed normal cartilage by the eighth decade. For the ankle, however, approximately 50% of joints had completely normal-looking cartilage through the sixth decade. Erosion of 30% or less of the cartilage surface (grade 3) began as early as the fourth decade for knees and the fifth decade for ankles. Diffuse cartilage erosion (grade 4) began as early as the fourth decade for knees, but only surfaced in three donor ankles and not until the eighth decade. Through the eighth decade, the majority (63%) of ankles still displayed either normal or fibrillation cartilage, whereas at this point, the majority of knees displayed moderate to severe degeneration.

**Figure 4:**
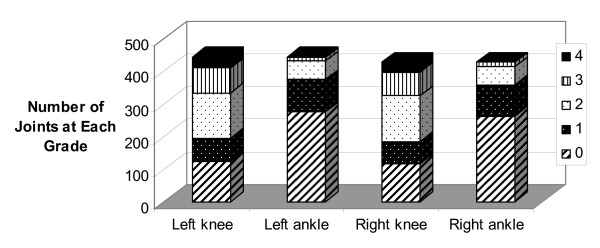
**Distribution of grades for the left and right knees and ankles separately**.

Knee and ankle cartilage scores, separately, for left and right sides are shown in Figure 4. There were no significant differences between sides (*P *> 0.05). The majority of individuals had more severe knee OA than ankle OA (60.8% on the left side and 60.5% on the right side). Fewer individuals had equal severity of OA on the knee and the ankle (30.1% on the left side and 36.9% on the right side). Rarely did the ankle display more severe OA than the knee (1.1% on the left side and 2.6% on the right side).

Results of the Wilcoxon signed rank test revealed that the only joint relationships which showed no significant differences were the left vs. right ankles and left vs. right knees (*P *= 0.1705 and 0.0845, respectively).

**Figure 5:**
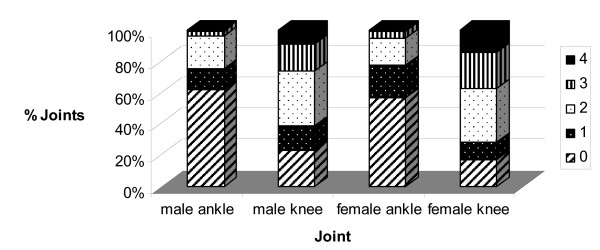
**Distribution of grades for male and female knees and ankles separately**.

Figure 5 shows cartilage scores by gender, where it can be seen that slightly fewer female knees and ankles were normal (grade 0) than male knees and ankles (*P *= 0.05). Females also showed more severe knee degeneration (grades 3 and 4) than did males (*P *= 0.03).

**Figure 6:**
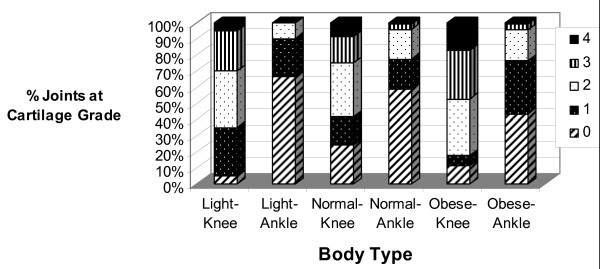
**Distribution of knee and ankle grades separated according to relative body type (as assessed visually)**. Knees from obese donors displayed more severe degeneration (grades 3 and 4) than did joints from lightweight or normal-weight donors.

The effect of weight on joint degeneration was significant (Figure 6). The knees of obese individuals showed more severe (grades 3 and 4) degeneration than did knees from normal-weight or lightweight donors (*P *< 0.05). For ankles, however, although there were more grade 0 joints in the lightweight category of donors, the differences at the more severe levels of degeneration that existed between obese and normal-weight donors for the knee did not exist for the ankle (*P *> 0.05 for grades 3 and 4).

The relationship between knee and ankle degeneration within individual donors can be resolved in several ways. An examination of which joint displays more severe degeneration than its counterpart within an extremity of an individual shows that the majority (61%) of donors had more severely degenerated knees than ankles on both left and right sides. Fewer (38.1% [left side], 36.9% [right side]) had knees and ankles of the same grade of degeneration, and only 1.1% (left side) and 2.6% (right side) had ankles which were more degenerated than knees. This was true for both left and right extremities. Ninety-nine percent of donors with normal (grade 0) knees also had normal ankles, whereas 38% of donors with normal ankles also had normal knees.

For an in-depth look at the most severe degeneration, Table 1 shows the percentage of ankle joints at each cartilage grade when the ipsilateral/contralateral knee joint displayed grade 4 degeneration, and vice versa for the opposite joint. From this table it is apparent that individuals with severely degenerated knees can have normal ankle cartilage; this was the case as often as their having fibrillated or fissured ankle cartilage. However, far fewer ankles displayed the same severity of degeneration as the ipsilateral or contralateral knee, although this condition did indeed exist. Although there were only three ankles displaying grade 4 degeneration, they were associated with degenerated knees.

**Table 1:** Percentage of ankle joints at each cartilage grade when the knee joint displayed grade 4 degeneration (the most severe degeneration)

*When knee cartilage grade = 4 (N = 54)*					
**Ankle Grade**	**0**	**1**	**2**	**3**	**4**
% of ipsilateral ankles	25.6	27.9	25.6	16.3	4.6
% of contralateral ankles	29.6	27.9	22.2	16.6	3.7
*When ankle cartilage grade = 4 (N = 3)*					
**Knee Grade**	**0**	**1**	**2**	**3**	**4**
% of ipsilateral knees	0	0	0	0	100
% of contralateral knees	0	0	0	33.3	66.7

Looking at the relationship between joint degeneration as the lower/higher joint of the limb changed in health status, Table 2 shows the distribution of matched and unmatched ankle grades within a donor with different levels of knee degeneration. Ankles within a donor increasingly did not match (i.e., did not have the same grade) as the grade of degeneration in the left knee increased through grade 3, with somewhat of a decline at grade 4 (*P *< 0.05 between all grades). For the right knee, there was an increase in the number of unmatched ankle grades as the knee grade increased through grade 2, at which point the percentage of unmatched grades was basically maintained (*P *< 0.05 between grades through grade 2; no significant difference between grades 2, 3 and 4). These data show the greater variability in joint health within individuals as disease progressed from normal to early signs of joint degeneration.

**Table 2:** Distribution of matching and unmatching ankles in association with the different levels of joint degeneration within the knee of the same donor

**Left Knee grade**	**0**	**1**	**2**	**3**	**4**
% ankle grades that match	95.8	88.0	78.4	70.6	82.8
% ankle grades that do not match	4.2	12.0	21.6	29.4	17.2
**Right Knee grade**	**0**	**1**	**2**	**3**	**4**
% ankle grades that match	94.4	56.2	30.8	36.2	33.3
% ankle grades that do not match	5.6	43.8	69.2	63.8	66.7

If both knees displayed grades of 3, 63.8% (30 of 47) of these ankles had the same grade. If both knees displayed grades of 4, 81.8% (18 of 22) of these ankles had the same grade. There were 11 donors (23.4% of grade 3 donors) in whom both knees were grade 3 and both ankles were grade 0. There were five donors (22.7% of grade 4 donors) in whom both knees were grade 4 and both ankles were grade 0.

**Figure 7:**
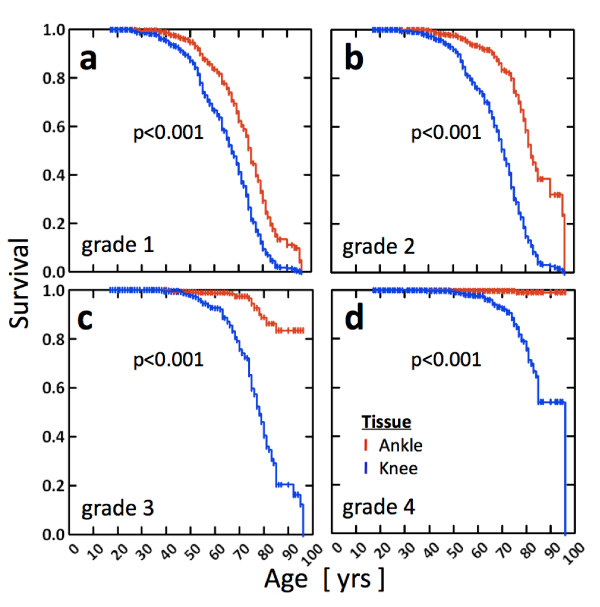
**Survival curves of the ankle (red) and the knee (blue), reflecting the probability of samples attaining grades (a) 1, (b) 2, (c) 3 or (d) 4**. There is a significant difference between expected survival of knees and ankles (*P *= 0.001).

Survival analysis to compare ankles and knees (Figure7) suggested an increasing incidence of degeneration with aging for both knees and ankles, yet markedly different survival curves for the ankle as compared to the knee (*P *< 0.001, red vs. blue lines, respectively) at all grades (1 to 4; Figures 7a-d). Mild degeneration (i.e., grade 1 or greater; Figure 7a) occurred at an earlier age for the knee; the mean survival ages for the knees and the ankles were ~65 and ~75 years, respectively. The gap in the mean survival age increases for moderate degeneration (grade 2 or greater; Figure 7b) as the mean survival ages for the knees and the ankles become ~70 and ~85 years, respectively. Severe degeneration (grade 3 or greater; Figure 7c) is found in ~80% of the knees by the age of 90 years, the age at which only <20% of the ankles were found to be equally degenerate. Grade 4 degeneration (Figure 7d) was found in ~50% of the knees by the age of 90 years, while only three of the ankles had grade 4 degeneration in the late decades.

**Figure 8:**
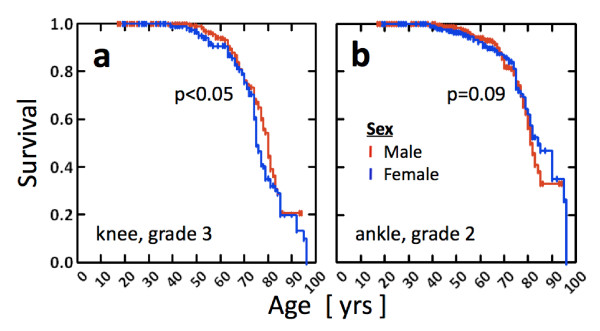
**Survival curves of the male (red) and female (blue) samples in **(a) **the knees reaching the grade 3 and **(b) **the ankles reaching the grade 2**. There were significant differences between the curves of the male and female knees when grade 3 (Figure 8a) or 4 was defined as being degenerate (for each *P *< 0.05)

Survival analysis to evaluate the sex differences (Figure 8) suggests significant differences between the survival curves of the male and female knees when grade 3 (Figure 8a) or 4 was defined as being degenerate (for each *P *< 0.05). This was due to a relatively delayed degeneration of the knee in males between 70 to 85 years of age. In the ankle, sex had no significant effect on survival curves, regardless of the grade (each *P *> 0.1). However, there was a trend (*P *= 0.09) of relatively delayed degeneration (to grade 2) in ankles of females between 80 to 95 years of age (Figure 8b).

## Discussion

OA is a condition based on both degenerative cartilage and bone changes within a joint resulting in the clinical manifestation of these changes as joint pain. In the present study, because the pain history of the individuals within our donor population was not available, we do not use the term *osteoarthritis*, but rather *joint degeneration *[20]. This is of significance because it is well known that some individuals with joint pain show no radiographic or magnetic resonance imaging evidence of joint disease, whereas other individuals with no joint pain show imaging evidence of the pathological joint changes normally associated with OA [20,21]. A strength of the present study, however, is that we had the advantage of actual visualization of articular cartilage surfaces and osteophytes from cross-sectional cadaveric donors, thus rendering data on normal and early stages of the disease which cannot be discerned through any current imaging technologies.

In a very early analysis of our donor population, when only 50 knee/ankle donor pairs had been harvested, we found that ankle joint degeneration was more frequent in men than in women, increased with age, and occurred most often in both limbs with the same severity [17]. In donors with degeneration in the ankle, the knee also showed degenerative changes with an equal or higher grade. At that time, we suggested that factors such as altered mechanics might be responsible for degeneration in one limb and result in changes in the contralateral limb. The present study on 545 knee/ankle donors with a mean age of 60 years reaffirms our previous results. For the knee joint, females showed greater degeneration than did males, with fewer normal joints and more joints displaying partial and extreme erosion of the articular surface. This concurs with the known greater prevalence of OA in women as compared with men [22,23]. This difference may be due to one or more of several known gender differences involving knee joint anatomy, kinematics and/or physiology [24-27]. One difference that we found in comparison to a previous study [16] was that here female donors had slightly less normal ankle cartilage and slightly more fibrillation than did males. However, this did not extrapolate to higher grades of degeneration, where male ankles displayed slightly earlier fissuring (grade 2) than did female ankles.

The effect of weight on joint degeneration was joint-specific whereby weight had a significantly greater effect on the knee than on the ankle. The majority of knees from obese donors displayed degeneration of at least grade 2 (fissuring) or greater, and nearly 50% displayed cartilage erosion down to subchondral bone. In the ankle, although lightweight donors displayed little fissuring and no erosion, the levels of fissuring and erosion were not different between normal-weight and obese individuals.

We found that approximately 20% of donors in whom both knees displayed advanced degeneration (grades of 3 or 4) had ankles that appeared perfectly normal; the reverse never occurred. This reinforces the idea that knee degeneration likely has a greater influence on ankle health than the reverse situation. The fact that knees may be bilaterally severely pathological in structure in the absence of visible ankle pathology attests to the structural stability of the ankle as a hinge joint with less mechanical freedom in comparison to the knee. It appears that, at least in some individuals, aberrant knee structure and function do not inevitably lead to changes in extremity function so severe that they affect the ankle. On a purely speculative level, however, it is likely that this protection would not be observed at the hip as the hip is much more highly prone to OA than the ankle, and the coexistence of hip and knee OA is well documented [28,29]. Survival analyses suggested that even mild degeneration (grade 1) occurs more slowly in the ankle than in the knee, and severe (grades 3 and 4) degeneration rarely occurs in the ankle. In addition, the effect of sex on joint degeneration was joint-specific and dependent on the severity of degeneration. In the knee, mild-to-moderate degeneration (grades 1 and 2) occurred similarly in both sexes; however, severe degeneration (grades 3 and 4) occurred at an earlier age for women. The trend reversed in the ankle.

Additionally, we explored the data a bit differently to further elucidate the relationship between the two joints within an extremity. One interesting relationship occurred when looking at how degeneration at the knee related to the matching of ankle grades within an individual. Ankle grades increasingly did not match within a donor as the grade of joint degeneration within the left knee increased through grade 3 (partial erosion of the articular surface), with somewhat of a decline at grade 4 (severe erosion). There was an increase in the number of unmatched ankle grades as right knee degeneration increased through grade 2 (fissuring), at which point the percentage of unmatched grades was basically maintained. This points to the greater variability in joint health within the extremities and results in an imbalance in joint health between sides as disease progresses. Combined with the finding that as degeneration in the knee increased so did degeneration in the ankle, an interesting consideration appears. Ninety-nine percent of donors with normal (grade 0) knees also had normal ankles, whereas 38% of donors with normal ankles also had normal knees. However, once signs of knee degeneration occur, even at the earliest stages (i.e., fibrillation), the ankles of a pair begin to become discordant in their appearance with respect to each other. We interpret this as suggesting that whatever mechanism is occurring in the knee to cause early degeneration, the same mechanism is likely occurring in the ankle, but at a lower level. This may be either as a consequence of mechanical alterations in the knee or through an independent process. It is likely, however, that the two are related as has been suggested in studies that have attempted to elucidate the relationship between knee and ankle OA.

Studies in patients have shown that hip-knee-ankle alignment contributes to the distribution of load across a joint surface. In fact, both the varus and valgus malalignment of the knee increase the risk of progression of medial and lateral OA, respectively [30,31]. The varus knee increases the force across the medial knee compartment, whereas the lateral compartment has increased force in the valgus knee [32]. In both these conditions, the mechanical alignment of the extremity is changed from the neutral axis, thus setting up alignment issues throughout the extremity and perhaps the entire body.

In a retrospective study of mechanical axis radiographs of subjects just prior to total knee arthroplasty, it was found that ankle OA and tilt in the ankle were not uncommon [16]. Furthermore, the greater the tilt in the ankle, the more degenerative were the changes in the joint [16]. When the mechanical axis at the knee was corrected at the time of surgery, the ankle tilt was also significantly changed.

This work relates well to one of our previous studies in which we found that the trabecular angle within the talar dome is associated with the level of joint degeneration [33]. The talar dome of the human talus receives compressive forces that have traversed the leg. Thus, in keeping with Wolff's Law, the body of the talus has predominantly vertically aligned trabeculae running superior to inferior. Through fast Fourier transform analysis, it was found that as the trabecular angle deviated from a perpendicular alignment, the greater were the cartilage changes on the articular surface, particularly at medial and lateral borders. We hypothesized that these results may be a reflection of the alignment and/or biomechanics at the joint [33]. Thus, taking the ideas of these latter two studies together, it is possible that a malaligned knee affects the alignment of the entire kinetic chain, setting the stage for potential pathology anywhere along that chain.

Another relationship that would have been interesting to examine is how medial vs. lateral knee OA is related to medial vs. lateral ankle OA. Unfortunately, because we did not have information on the topographical location of cartilage changes, we cannot make any statements in this regard. However, in a previous cadaveric study, we found that more knee and ankle joints displayed greater degeneration on the medial than on the lateral aspect [17]. In another study, on the difference between foot center of pressure patterns between subjects with and without OA, we found that the subjects with medial compartment OA demonstrated a more laterally placed foot pressure pattern with normal walking as compared with non-OA control subjects [34]. This is accomplished by changing the axis of the ankle joint in relation to the leg and placing greater pressure on the medial aspect of the ankle. Therefore, at least from these results, it might be expected that medial ankle OA could be found in relation to knee OA. However, further studies must be carried out to make this determination.

The limitations of the present study include the lack of information on the history of joint injury and the lack of information on the level of mobility or the use of walking aids. Each of these issues has the potential to introduce variability in the data that might not be accounted for. For instance, if a subject sustained an undocumented traumatic injury to the knee joint, it would not be known if the presence of OA in this joint was due to trauma or to the relationship of this joint to the contralateral knee or the ankles. Another limitation is that we did not have access to the distal tibia. If the joint degeneration on this component is greater than that on the talus of the same joint, this may lead to the underestimation of the true severity of ankle pathology. This would surely be the case in at least some specimens, as we previously showed in a sample of 100 specimens from 50 cadavers that 30% of ankle joints displayed greater degeneration on the tibia than on the talus, 21% showed equal levels of degeneration on both sides and 49% showed greater degeneration on the talus [18].

Another parameter of consideration is the manner in which body type (light, normal, obese) was determined. Because we obtained the joints through the Gift of Hope Organ and Tissue Donor Network, we were dependent upon subjective determination after physical examination of the body. We considered the amount of overall subcutaneous body fat in making this determination, and although not entirely scientific, we think this method provides good relative information within the study sample.

## Conclusions

To our knowledge, this cadaveric donor joint study is the largest study of its kind for knee and ankle pathology. The knee joint displayed significantly greater signs of degeneration than the ankle joint and showed a gender preference whereby females had more severe knee degeneration. Obesity increased the severity of joint lesions in the knee but had a much less profound effect on the ankle. A major new finding of the study was that ankle grades increasingly did not match within a donor as the grade of joint degeneration in either the left or the right knee increased. This is, in essence, an imbalance in joint integrity between sides and points to a greater variability in joint health within the extremities as disease progresses. The possibility of this leading to limb malalignment, particularly once cartilage erosion in at least one knee compartment has occurred, is realistic. In turn, limb malalignment is highly associated with joint disease progression as shown in other studies.

## Competing interests

The authors declare that they have no competing interests.

## Authors' contributions

CM analyzed the data and wrote the manuscript. AM harvested and graded the cadaveric human knees and tali from 2004 through 2009. WB performed the survival analysis, prepared some of the figures and contributed to the manuscript writing. KM performed statistical analyses and contributed to the manuscript writing.

## Pre-publication history

The pre-publication history for this paper can be accessed here:

http://www.biomedcentral.com/1741-7015/8/48/prepub
